# Obtaining and Studying the Properties of Composite Materials from *ortho*-, *meta*-, *para*-Carboxyphenylmaleimide and ABS

**DOI:** 10.3390/molecules31010190

**Published:** 2026-01-05

**Authors:** Eldar Garaev, Shahana Guliyeva, Aygun Alikhanova, Konul Huseynguliyeva, Bakhtiyar Mammadov

**Affiliations:** 1 Department of Pharmaceutical Toxicology and Chemistry, Faculty of Pharmacy, Azerbaijan Medical University, Baku AZ1022, Azerbaijan; 2 Institute of Polymer Materials of the Ministry of Science and Education of the Republic of Azerbaijan, Sumgait AZ5004, Azerbaijan

**Keywords:** *o*-, *m*-, *p*-carboxyphenylmaleimide, ABS, composite materials, antibacterial properties, thermal stability, rheological parameters, polymer modifiers

## Abstract

This work presents the results of the synthesis and investigation of new antibacterial composite materials based on acrylonitrile–butadiene–styrene (ABS) copolymer and *o*-, *m*-, *p*-carboxyphenylmaleimides (CPhMI). The composites were obtained by thermal mixing with varying contents of different CPhMI isomers in the polymer matrix. The structural and thermal characteristics of the synthesized materials were investigated using IR and UV spectroscopy, as well as thermogravimetric (TGA) and differential thermal analysis (DTA). The results indicate that the *o*-isomer imparts the highest thermal stability, while the *p*-isomer shows slightly lower stability. In terms of processability, the *m*-isomer exhibits the highest melt flow, the *p*-isomer an intermediate level, and the *o*-isomer the lowest. The antibacterial activity of the composites was evaluated by the agar diffusion method against Gram-positive (*Staphylococcus aureus*) and Gram-negative (*Escherichia coli*) microorganisms. All synthesized samples exhibited strong antibacterial activity against *S. aureus* and *E. coli* at a concentration of 0.5 wt%, confirming their potential for application in medical devices, as well as in sanitary polymer coatings and packaging.

## 1. Introduction

Demand for antimicrobial materials is growing rapidly, especially in the healthcare, food safety, and consumer goods manufacturing sectors, where microbial contamination can harm human health and reduce product quality [1,2,3,4,5]. The housings, cases, and polymer coatings of medical devices such as blood pressure monitors, thermometers, inhalers, nebulizers, and others, when repeatedly used in outpatient clinics, eventually become a source of infection.

Acrylonitrile butadiene styrene (ABS) is a versatile thermoplastic polymer widely used in mechanical engineering, aviation, pharmaceuticals, medicine [6], and household appliances [7] due to its excellent mechanical strength, impact resistance, and ease of processing. However, in environments where hygiene is crucial, such as medical facilities, food packaging, and household appliances, conventional ABS is not resistant to microbial contamination. To overcome this limitation, researchers have developed composite materials (CM) based on ABS with the addition of antibacterial agents that can effectively suppress the growth of pathogenic microorganisms. Furthermore, these advanced composites not only retain the desirable structural and technological properties of ABS, but also provide increased safety, durability, and functionality, making them highly valuable for applications that require both the necessary mechanical characteristics and antimicrobial protection [8,9,10]. Recently, efforts have focused on improving the properties of ABS using high-molecular-weight antimicrobial additives. Antibacterial composites made from ABS plastic can be used in the manufacture of medical equipment, protective devices, and on surfaces in hospitals to reduce the risk of infection [11,12,13].

Polymer materials are often subject to biocorrosion. In this case, they are damaged by microfungi; bacteria absorb additives in plastic products, reducing their quality and service life, thereby turning them into a source of infection. To prevent this, natural and synthetic biologically active additives (BAA) are introduced into polymer composite materials during the extrusion process. Currently, there are many known biocidal additives. However, strict requirements for polymer additives, such as relatively high heat resistance, toxicity, miscibility with other components, and many others, limit their application [14,15,16,17,18,19,20].

Commonly used polymers are typically employed as a matrix for obtaining bactericidal polymer compositions [21]. Silver metal powder and silver salts are widely used as antimicrobial additives to polyolefins, polystyrene, styrene copolymers, polyamides, and their mixtures with polyethylene and ABS, despite their high cost [22,23].

A common drawback of most analogs and composite materials is the gradual loss of their bactericidal and fungicidal properties as a result of surface leaching of the biocidal ingredient [24,25,26,27].

The aim of the study was to develop antibacterial composite materials that possess favorable polymer properties for use in medical devices.

The objective of the research is to obtain new antibacterial composite materials based on ABS with a broad spectrum of antibacterial activity, using compounds containing imide and carboxyl groups in the macromolecule, in particular CPhMIs as biologically active additives [28].

## 2. Results

### 2.1. Identification of Compositions of o-, m-, p-Carboxyphenylmaleimide/ABS

**Figure 1:**
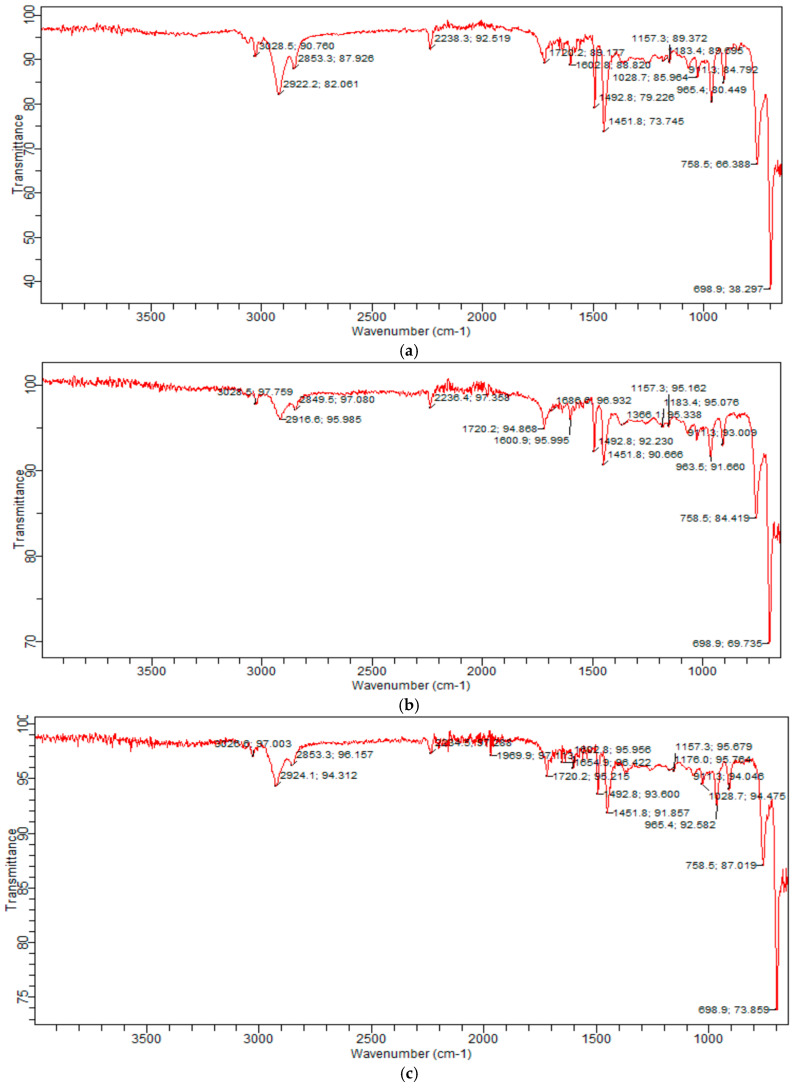
IR spectrum of the composition *o*- (**a**), *m*- (**b**), *p*-carboxyphenylmaleimides (**c**)/ABS.

Based on the results of IR spectroscopy, the composition of the composite materials are shown in Figure 1.

The following absorption bands were observed in the IR spectrum: deformation vibrations of the C–H bonds of the CH_2_ and CH_3_ groups at 1451 cm^−1^, valence vibrations of the C–H bonds at 2851 and 2918 cm^−1^, deformation vibrations of the C–H bond of the m-substituted benzene ring at 758 cm^−1^; valence vibrations of the C–C bond of the benzene ring at 1602 cm^−1^, the C–O bond at 1028 and 1157 cm^−1^, and the C=O bond at 1720 cm^−1^ [29,30,31]. Pure ABS plastic has a characteristic absorption band at 698 cm^−1^ for the –CH_2_ vibration, while the band at 2235 cm^−1^ was due to the -R-C≡N group.

**Figure 2:**
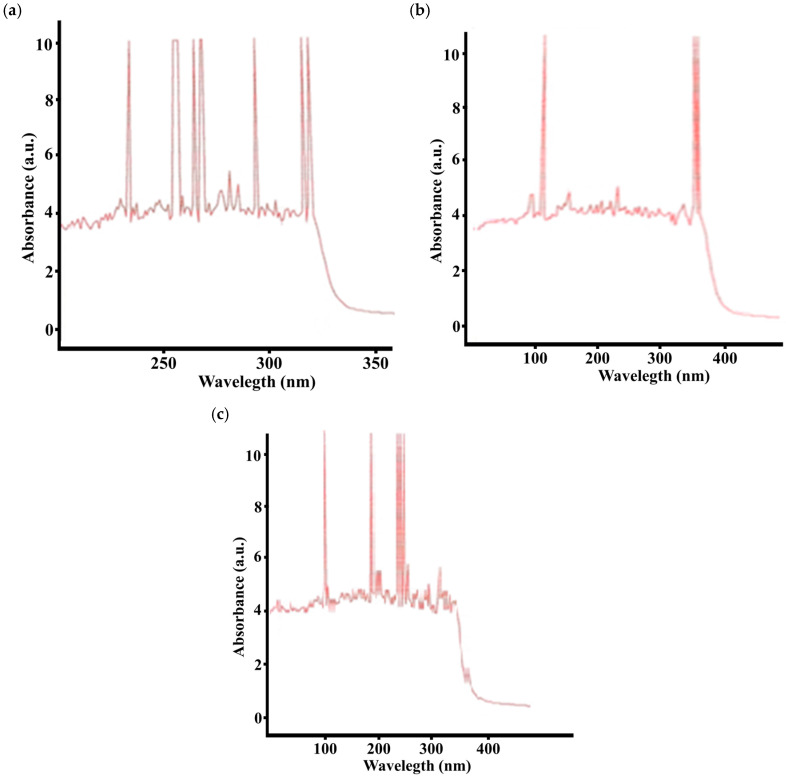
UV spectrum of the composition *o*- (**a**), *m*- (**b**), *p*-carboxyphenylmaleimides (**c**)/ABS.

Based on the results of UV spectroscopy, the composition of the composite materials are shown in Figure 2.

To study the electronic transitions in the synthesized *o*-, *m*-, and *p*-isomers of CPhMI, UV spectra were recorded. The spectra of all samples exhibit characteristic absorption bands due to π→π* and n→π* transitions in aromatic and imide conjugated systems.

For the *o*-isomer, intense absorption bands appear at 228–235 nm and 242–250 nm, corresponding to π→π* transitions in the butadiene and phenyl moieties. In the 258–270 nm region, a π→π transition of the maleimide unit is observed, along with the onset of an n→π* transition of the C=O bond. Bands at 275–290 nm are associated with the n→π* transition of the imide carbonyl, with only a minor influence from the nitrile group. In the 295–305 nm range, slight band shifts may occur due to intramolecular hydrogen bonding. In the range of 300–320 nm, a hybrid π→π*/n→π* transition of the conjugated aromatic-imide system is observed, followed by a sharp decrease in intensity (320–340 nm).

The *m*-isomer is characterized by absorption bands at 225–232 nm (π→π* transitions of butadiene and styrene fragments) and 240–248 nm (phenyl system). In the 250–265 nm region, a π→π* transition of the maleimide group is observed; due to weaker resonance interaction, the peaks are shifted to the short-wave region. At 275–285 nm, an n→π* transition of the imide fragment is recorded.

The *p*-isomer is characterized by bands at 230 nm (π→π* transition of butadiene and phenyl fragments), 245 nm (π→π* transition of the phenyl link), and a narrow peak at about 255 nm associated with an aromatic or maleimide π→π* transition. In the 280 nm region, an n→π* transition of the maleimide carbonyl group and a weak influence of the nitrile group are recorded. The band at 295–300 nm is due to the hybrid transition of the conjugated aromatic-imide system, followed by a sharp decrease in intensity (320–340 nm).

The *o*- and *m*-isomers are characterized by a stable absorption zone of 270–285 nm, corresponding to the n→π* (C=O) transition and the influence of the nitrile group. For the p-isomer, the main stable region is 280–295 nm, which indicates more pronounced conjugation and interaction between the aromatic and imide systems.

The general region of intense absorption for all isomers is 200–320 nm, typical for polymers with conjugated aromatic-imide chains.

### 2.2. Physical and Mechanical Parameters of CM Based on o-, m-, p-CPhMI/ABS

Changes in the physical and mechanical properties of ABS-based composite materials during extrusion with the addition of biologically active additives (BAA) based on 0.50 ÷ 3.0% carboxyphenylmaleimides are shown in Table 1.

As can be seen from Table 1, the physical and mechanical properties of composite materials containing 0.50% BAA (carboxyphenylmaleimides) change only slightly compared to the original ABS, which is likely due to the organic nature and low amount of bactericidal additives. All physico-mechanical measurements were carried out in four independent replicates (*n* = 4), and the reported values correspond to the mean of these measurements.

### 2.3. Antibacterial Evaluation

**Figure 3:**
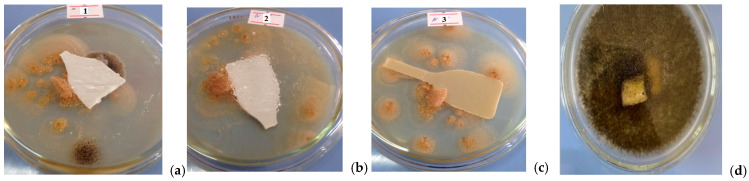
Antibacterial activity of the composition *o*- (**a**), *m*- (**b**), *p*-carboxyphenylmaleimides (**c**)/ABS and neat ABS (**d**).

The antimicrobial activity of the synthesized carboxyphenylmaleimide monomers was evaluated using the disk-diffusion method. The compounds showed significant effects against isolated microbial cultures in concentrated form, while their aqueous solutions exhibited only weak activity, with the strongest inhibition observed against *Candida albicans*. The resistance of ABS-based composites containing these additives was also assessed against microfungi (*Aspergillus niger*, *A. ochraceus*, *Penicillium cyclopium*, *Cladosporium herbarum*, *Fusarium moniliforme*, and *F. oxysporum*) over a one month period in a nutrient medium. Except for unmodified ABS, no fungal colonization or surface degradation was observed, based on visual inspection (Figure 3).

In summary, while the antimicrobial activity is primarily attributable to the pure carboxyphenylmaleimide compounds, ABS composites containing small amounts of these additives effectively resist fungal growth in the solid state, and their incorporation has minimal impact on the physical and mechanical properties of the materials, supporting their potential application in practical settings.

### 2.4. In Silico Studies

Computer studies were conducted using recognized online prediction platforms to obtain preliminary information about the biological activity and safety profile of the initial low-molecular-weight compound. Potential antimicrobial activity was assessed using the PASS Online server (Way2Drug, https://www.way2drug.com, accessed on 18 November 2025), which uses structure-activity relationship models to predict possible biological effects based on chemical structure similarity. The predictions provided qualitative estimates of the likelihood of interaction with various types of microorganisms and were used for comparative analysis of the compound’s isomers. Toxicological properties were further evaluated using the ProTox-3.0 platform (https://tox.charite.de/protox3, accessed on 18 November 2025), which predicts general toxicity endpoints based on molecular structure and machine learning models. In addition, carcinogenicity was assessed using the CarcinoPred-EL (http://112.126.70.33/toxicity/CarcinoPred-EL/result/?id=W9nk0Y4K3oTGql8OoXzc, accessed on 18 November 2025) web server, which uses ensemble learning algorithms, including random forest, support vector machine, and XGBoost models, in combination with several molecular descriptors (CDK, CDKExt, CDKGraph, KR, KRC, MACCS, and PubChem fingerprints). The results of the calculations were interpreted qualitatively and used as supplementary information to complement the experimental results, rather than as definitive evidence of biological efficacy or toxicity.

The in silico toxicity assessment performed using the ProTox-3.0 platform [32] predicted an LD_50_ value of 710 mg/kg for oral administration for the test compound, which corresponds to toxicity class 4 according to the Globally Harmonized System. This classification indicates a moderate level of acute toxicity. The prediction was obtained with an average structural similarity of 68.83% to compounds in the reference dataset and an overall prediction accuracy of 68.07%, indicating reasonable model reliability.

These results indicate that this compound is unlikely to pose a significant risk of carcinogenicity or environmental toxicity, confirming its potential suitability for use in polymer or biomedical composite materials.

### 2.5. Study of Dynamic and Kinematic Viscosity of the Composition

A comparative study of the dynamic and kinematic viscosity of composite materials based on *o*-, *m*-, *p*-CPhMI/ABS was conducted over a range of temperatures following the methodology described in [33,34].

A comparative study of the dynamic and kinematic viscosity of composite materials based on *o*-, *m*-, and *p*-CPhMI/ABS was conducted over a temperature range of 15–45 °C (Table 2). All measurements were performed in triplicate.

As the temperature increases, all the samples studied exhibit a consistent decrease in dynamic viscosity, which is associated with increased macromolecular mobility and the weakening of intermolecular interactions in the polymer matrix. The composition of *p*-CPhMI/ABS occupies an intermediate position in terms of viscosity, while *o*-CPhMI/ABS exhibits the highest values, which indicates, in this case, a denser packing of macromolecules and the presence of additional intermolecular bonds.

**Figure 4:**
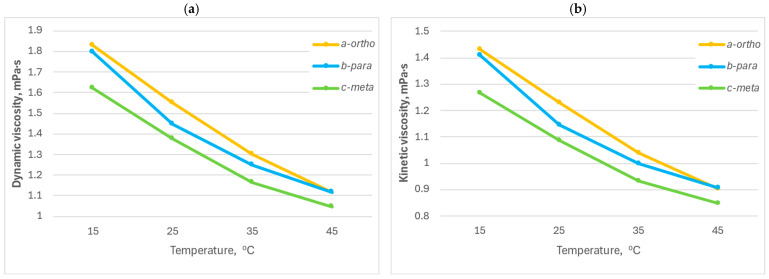
Dynamic (**a**) and kinematic (**b**) viscosity of the composition *o*-, *m*- and *p*-carboxyphenylmaleimides/ABS at different temperatures.

A similar trend can be observed in the graphs showing the dependence of kinematic viscosity on temperature (Figure 4): when heated, the viscosity of all samples of the composition decreases, but the relative ratio between the isomers added to the composition remains unchanged. The most fluid samples are those containing the *m*-isomer, which has the lowest steric hindrance and the weakest intramolecular interactions. Samples obtained with the *p*-isomer demonstrate an average viscosity value among the compositions obtained, which may be due to a more symmetrical structure and a moderate degree of interaction between the aromatic and imide fragments. While compositions obtained with the o-isomer are characterized by the highest viscosity, which is explained by the possible formation of intramolecular hydrogen bonds between the carboxyl and imide groups, as well as the restriction of chain mobility due to spatial factors.

Thus, the data obtained indicate that the rheological behavior of the composites under study directly depends on the position of the substituents in the phenyl ring of the carboxyphenylmaleimide fragment. The isomeric structure determines the degree of conjugation and intermolecular interactions during the formation of the composition and, as a result, the viscosity characteristics of the resulting composite materials.

### 2.6. Study of Thermal Properties of the Composition

The results of the Differential Thermal Analysis (DTA) and Thermogravimetric Analysis (TGA) of the obtained composite materials based on *o*-, *m*-, *p*-CPhMI/ABS have also been determined [35,36].

**Figure 5:**
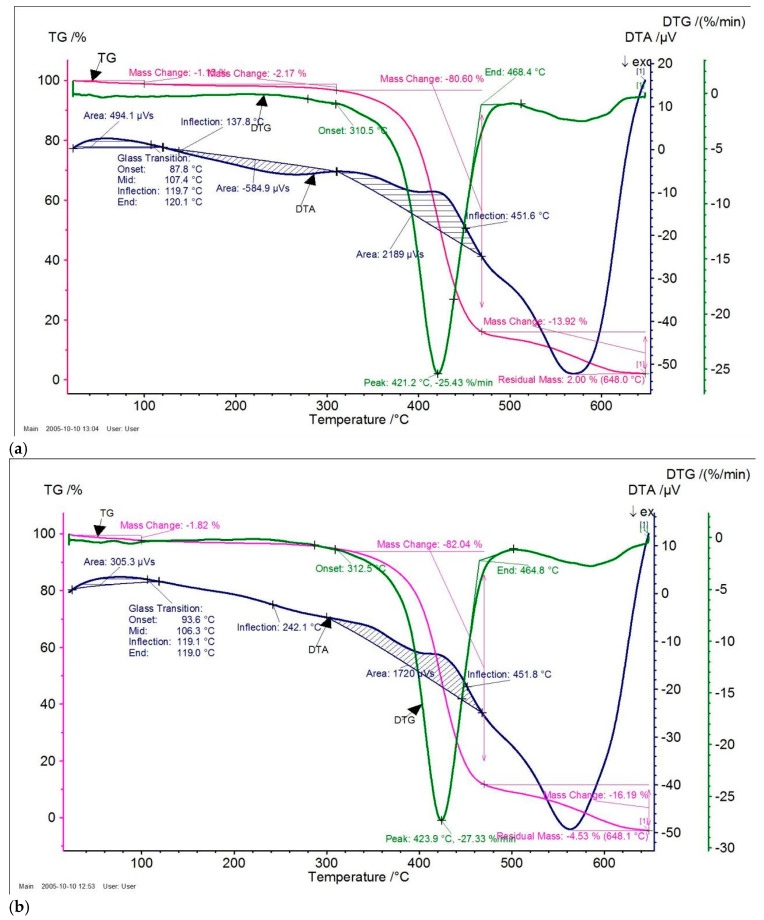

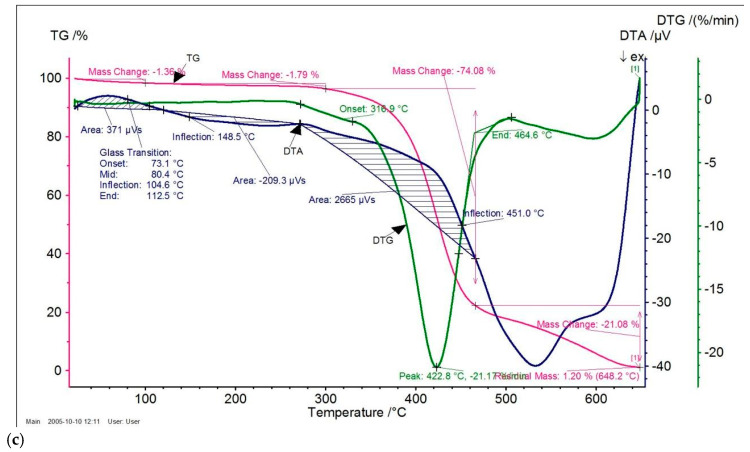
Thermogravimetric (TG) and Differential Thermal Analysis (DTA) of *o*- (**a**), *m*- (**b**), and *p*- (**c**) carboxyphenylmaleimides/ABS Compositions.

The thermal behavior of the material was investigated using thermogravimetric analysis (TGA), derivative thermogravimetry (DTG), and differential thermal analysis (DTA) (Figure 5). The TGA curve (pink) shows the change in sample mass as a function of temperature, providing information on thermal stability and decomposition stages; temperature intervals with insignificant mass loss indicate thermally stable regions, while sharp decreases correspond to decomposition processes. The DTG curve (green), representing the first derivative of the TG signal, reflects the rate of mass loss and allows the stages of degradation to be precisely determined, with a pronounced minimum observed in the range of approximately 422–451 °C corresponding to the main decomposition event. The DTA curve (blue) records the thermal effects associated with endothermic and exothermic processes; shifts in the baseline on this curve are explained by the glass transition temperature (Tg), which is not accompanied by mass loss, as confirmed by the constant TG profile in this region. Distinct DTA peaks indicate thermal or chemical transformations occurring during heating. The shaded areas under the DTA peaks represent the enthalpy change (ΔH) of the corresponding thermal events and are expressed as integrated areas (μV·s).

The activation energy was calculated using the double-logarithm method presented in the work (Table 3). The activation energy of thermal degradation was determined using the double logarithmic method based on thermogravimetric (TG) analysis [37]. The onset temperature of degradation (T_n_) was first identified from the TG curve. Subsequently, the mass loss of the sample (ΔG) was recorded at 10 °C temperature intervals above T_n_.

For each temperature interval i, the ratio of successive mass losses was calculated as:


$$\frac{∆G_{i}}{∆G_{i+1}}$$

The obtained ratios were subjected to double logarithmic transformation according to:


$$y=ln\left[ln(\frac{∆G_{i}}{∆G_{i+1}})\right]$$

The activation energy of degradation (*E_a_*) was calculated using the equation:


$$E_{a}=R\cdot \tan\alpha$$

The thermal stability of ABS composites with *o*-, *m*-, and *p*-carboxyphenylmaleimides showed that *o*- and *p*-isomers significantly increase thermal stability and glass transition temperature compared to pure ABS.

Although pure ABS was not investigated experimentally in this study, comparison with well-documented literature data indicates that the incorporation of *o*- and *p*-carboxyphenylmaleimide fragments into the ABS matrix results in an increase in the glass transition temperature and a shift in the main thermal decomposition stage toward higher temperatures. Thermal analysis of the ABS composites modified with *o*-, *m*-, and *p*-carboxyphenylmaleimides demonstrates that the *o*- and *p*-isomers provide a pronounced improvement in thermal stability and glass transition temperature relative to typical ABS systems, whereas the *m*-isomer exhibits a weaker stabilizing effect. These findings indicate an overall enhancement of the thermal stability of ABS composites upon incorporation of structurally favorable carboxyphenylmaleimide units.

The composite with *o*-CPhMI demonstrated the highest thermal stability (Tg ≈ 107 °C, decomposition peak ≈ 421 °C), while *p*-CPhMI improved carbon residue formation and polymer matrix rigidity, and the composition obtained with the m-isomer is characterized by a reduced Tg (≈80 °C) and minimal coke residue, indicating weak polymer matrix stabilization.

Thermal analysis of ABS-based composites modified with *o*-, *m*-, and *p*-CPhMI shows clear differences in thermal stability and glass-transition behavior.

The *o*-CPhMI/ABS composite demonstrated the highest thermal stability, with the main decomposition starting at approximately 325–330 °C, the DTG peak occurring at around 421 °C, and the average glass transition temperature (Tg) being 107 °C. The composite retained its structural rigidity and had a low coke residue (~2%), indicating effective stabilization of the polymer matrix.

The thermal properties of the *p*-CPhMI/ABS composite are also improved compared to pure ABS, exhibiting a Tg of 106 °C and a slightly lower decomposition onset temperature (~312 °C), with a carbon residue of (~4.5%).

The *m*-CphMI/ABS composite demonstrated the lowest thermal stability: Tg 80 °C, thermal decomposition range ~317–423 °C, and minimum residue (~1.2%).

## 3. Materials and Method

### 3.1. Chemicals

In order to obtain antibacterial ABS compositions characterized by a broad spectrum of biological activity, 0.5% (by weight) of CPhMI isomers were introduced into the composition of the materials during the processing stage [38]. The composition of the initial ABS compositions included 70 wt% ABS and 30 wt% magnesium hydroxide (Mg(OH)_2_).

When preparing ABS-based composite materials, four samples with different compositions were formed. Sample CM_1_ contained 70 wt.% ABS, 30 wt.% magnesium hydroxide (Mg(OH)_2_) and 0.5 wt.% *o*-CPhMI. In sample CM_2_, 0.5% by mass of *m*-CPhMI was used instead of the *o*-isomer, and sample CM_3_ contained 0.5% by mass of *p*-CPhMI. Control sample CM_4_ consisted of 70% by mass % ABS and 30 wt.% magnesium hydroxide without CPhMI additives. All ingredients were thoroughly mixed, then extruded at a temperature of 170–200 °C and pressed into standard plates under a pressure of 10–14 MPa.

### 3.2. Instruments

The prepared composite materials were characterized by using a variety of techniques. The IR spectra of the samples were recorded on an Agilent Cary 630 FTIR spectrometer (Agilent Technologies, Santa Clara, CA, USA) using a ZnSe crystal in the wavenumber range of 600–4000 cm^−1^. UV spectra were obtained in the range of 200–800 nm using an identical instrument setup.

The resulting mixtures were extruded using a WPM apparatus (VEB Thüringer Industriewerk Rauenstein; IIRT-Plastomer extruder, Rauenstein, Germany) at a temperature range of 170–200 °C. During the extrusion process, the materials were thoroughly homogenized, ensuring uniform dispersion of all additives within the ABS matrix, which is critical for achieving consistent physico-mechanical and thermal properties in the final composite. The density of the resulting composites was measured using an IIRT-M device, specifically designed for determining the density of polymer alloys. This instrument allows for precise and reproducible measurements, providing critical data for assessing the effect of additives on the composite’s structural compactness and overall material performance.

### 3.3. The Study of Antibacterial Properties of CM Based on o-, m-, p-CPhMI/ABS

The antimicrobial activity of synthesized monomers was evaluated using the disk diffusion method [39]. All microorganisms studied were examined in a vegetative (non-spore-forming) state, and experiments were conducted using actively growing cultures in the logarithmic phase. The microorganisms studied included *Staphylococcus aureus* (Gram-positive), *Escherichia coli* and *Pseudomonas aeruginosa* (Gram-negative), *Bacillus anthracoides* (gram-positive spore-forming bacterium), *Klebsiella pneumoniae* (encapsulated Gram-negative bacterium), and *Candida albicans* (yeast-like fungus).

Microbial suspensions were evenly inoculated onto nutrient agar plates, after which the test samples were applied. Antimicrobial activity was assessed by the formation and diameter of inhibition zones around the samples, reflecting their effect on the vegetative cells of bacteria and fungi.

### 3.4. Antibacterial Methodology

The disk-diffusion method was used to study the antibacterial and antifungal properties of maleimides. To evaluate antimicrobial activity, a range of microorganisms was used, including *Staphylococcus aureus* (Gram-positive), *Escherichia coli* and *Pseudomonas aeruginosa* (Gram-negative), *Bacillus anthracoides* (spore-forming Gram-positive), *Klebsiella pneumoniae* (capsular Gram-negative) and *Candida albicans* (fungus).

Microbial suspensions were prepared from fresh daily cultures by diluting approximately 1 mL of microbial cells in 1 mL of sterile physiological solution. Each suspension was evenly spread onto Petri dishes containing appropriate growth media—Meat-Peptone Agar (APA) for bacteria and Sabouraud Agar for fungi. Excess suspension was removed with a pipette and safely discarded in disinfectant. The inoculated plates were allowed to dry at 37 °C for 10 min.

Sterile disks, preheated on a sterile surface for 3–5 min, were placed gently onto the inoculated media using sterile forceps, ensuring good contact with the surface. Plates with APA were incubated at 37 °C, while those with Sabouraud medium were incubated at 28 °C. The active compounds diffused into the agar from the disks, inhibiting microbial growth. After 24–48 h of incubation, the plates were examined, and the zones of inhibition were recorded.

Three types of fungi were used in the study: *Aspergillus niger*, *Aspergillus ochraceus*, and *Fusarium nugamai*, grown on a nutrient medium with agar-saburo. The working chamber was pre-disinfected with alcohol, after which a UV-C germicidal lamp (254 nm) was switched on to maintain sterility. The culture medium was melted on a heating surface and poured into Petri dishes under an open flame to minimize contamination. Each dish was filled to approximately one-third of its volume.

After the medium had dried, the fungal mycelium was inoculated using sterile lancets, placing the plastic samples under investigation next to the cultures. Incubation was carried out at 28 °C for 1 month to study the effect of fungi on plastic.

Preparation of the medium: 65.5 g of agar-saburo was dissolved in 1 L of distilled water and autoclaved for 15 min at 1 atm. After autoclaving the medium was cooled to a temperature suitable for filling and filtration.

### 3.5. In Silico Studies

In silico studies were conducted using such software as PASS online v2.0 [https://www.way2drug.com, accessed on 18 November 2025], ProTox 3.0 [https://tox.charite.de/protox3, accessed on 18 November 2025] and CarcinoPred-EL (http://112.126.70.33/toxicity/CarcinoPred-EL/result/?id=W9nk0Y4K3oTGql8OoXzc, accessed on 18 November 2025). These analyses were performed to support the experimental investigation using established online prediction platforms. The antimicrobial potential of the initial low-molecular-weight compound was evaluated using the PASS Online (Way2Drug) server, which predicts biological activity based on structure–activity relationship (SAR) models. Predictions were generated for all positional isomers using default parameters. The potential carcinogenicity of monomers was assessed using the CarcinoPred-EL web server. This platform employs ensemble machine-learning approaches, including Random Forest (RF), Support Vector Machine (SVM), and XGBoost algorithms, in combination with multiple molecular descriptors, such as CDK, CDKExt, CDKGraph, KR, KRC, MACCS, and PubChem fingerprints. All calculations were carried out using the standard settings provided by the server. The in silico results were used for preliminary evaluation and comparative analysis and were intended to complement, but not replace, experimental biological and toxicological studies.

### 3.6. Study of Viscosity of the Composition

The viscosity of the samples under investigation was determined using an Anton Paar rotational viscometer, which operates based on measuring the torque required to rotate a spindle immersed in the liquid sample. Additionally, a Stabinger Viscometer SVM 3000 (Anton Paar GmbH, Graz, Austria) was used, which determines viscosity and density simultaneously using a combined rotational and oscillating U-tube measurement principle. The resistance to rotation is proportional to the viscosity, which makes it possible to accurately determine the rheological characteristics of the material at different shear rates and temperatures.

The dynamic and kinematic viscosities of composite materials based on *o*-, *m*-, and *p*-CPhMI/ABS were measured using an Anton Paar rotational viscometer (MCR series), following a methodology analogous to that reported in [33,34]. Samples were equilibrated at the target temperatures (15, 25, 35, and 45 °C) for at least 10 min prior to measurement and gently stirred to remove air bubbles. Dynamic viscosity was measured in rotational mode using a cone-and-plate spindle suitable for low-viscosity polymer composites, with shear rates varied according to the manufacturer’s recommendations to confirm Newtonian behavior. Kinematic viscosity was calculated from the dynamic viscosity and sample density (ν = η/ρ), with densities determined using a pycnometer at the corresponding temperatures. All measurements were performed in triplicate, and the mean values were reported; variability between repeats was within ±2%. The viscometer was calibrated prior to each measurement series according to standard procedures. The temperature-dependent viscosity data were analyzed to assess the effect of isomeric structure (*o*-, *m*-, *p*-) of the CPhMI fragment on macromolecular mobility and intermolecular interactions.

### 3.7. Thermal Analysis of the Composition (DTA/TGA)

Thermal analysis of the composites was carried out using a Netzsch STA 449 F3 instrument in the temperature range of 20–1650 °C at heating rates from 0.001 to 50 °C/min. The system provides a temperature control accuracy of ±0.3 °C, allows a maximum sample mass of up to 5 g, and offers a resolution of 1 µg. Measurements were conducted under controlled gas atmospheres (N_2_, Ar, O_2_, H_2_, or air) with flow rates between 5 and 250 mL/min. The thermal stability of the composites was evaluated using characteristic parameters T_0_, T_50_, and T_max_.

## 4. Conclusions

In this study, ABS-based composite materials modified with *o*-, *m*-, and *p*-carboxyphenylmaleimides were successfully prepared by thermal mixing. Spectroscopic analyses confirmed the formation of strong intermolecular interactions between ABS and the modifiers, while UV spectra revealed characteristic absorption bands typical of conjugated aromatic imide systems. Thermal analysis showed that the in-corporation of carboxyphenylmaleimides improves the thermal stability of ABS, with the *o*-isomer providing the highest glass transition temperature and thermal resistance. Rheological measurements indicated a decrease in viscosity with increasing temperature for all composites, with the *m*-isomer exhibiting the highest melt fluidity. All modified materials demonstrated pronounced antibacterial activity against *Staphylococcus aureus* and *Escherichia coli*, as well as antifungal activity against *Candida albicans*. These findings highlight the potential of the developed composites for applications in medical devices, hygienic coatings, and antimicrobial packaging.

## Figures and Tables

**Table 1:** Physico-mechanical parameters of the antibacterial CM based on *o*-, *m*-, *p*-carboxyphenylmaleimides/ABS.

Samples	BAA in the Composition of CM	Relative Elongation %	Mechanical Strength MPa	Melt Flow Index (MFI) g/10 min
CM_1_	*o*-CPhMI	40	37.6	1.41
CM_2_	*m*-CPhMI	40	37.7	1.45
CM_3_	*p*-CPhMI	41	38.7	1.44
CM_4_	ABS without additives	42	38.8	1.42

**Table 2:** Dynamic (a) and kinematic (b) viscosity of the CM based on *o*- (a), *m*- (b), *p*-carboxyphenylmaleimides (c)/ABS at different temperatures.

Temperature, °C	CM	Dynamic Viscosity, mPa·s	Kinematic Viscosity, mm^2^/s	Density, g/cm^3^
15	*o*-CPhMI/ABS	1.8287	1.4321	1.2769
	*m*-CPhMI/ABS	1.6193	1.2678	1.2773
	*p*-CPhMI/ABS	1.7974	1.4084	1.2762
25	*o*-CPhMI/ABS	1.5520	1.2291	1.2627
	*m*-CPhMI/ABS	1.3740	1.0881	1.2627
	*p*-CPhMI/ABS	1.4455	1.1458	1.2616
35	*o*-CPhMI/ABS	1.2981	1.0403	1.2479
	*m*-CPhMI/ABS	1.1651	0.93346	1.2481
	*p*-CPhMI/ABS	1.2464	0.99966	1.2468
45	*o*-CPhMI/ABS	1.1152	0.90448	1.2330
	*m*-CPhMI/ABS	1.0471	0.84889	1.2335
	*p*-CPhMI/ABS	1.1181	0.90758	1.2320

**Table 3:** Mass loss and activation energy during thermodestruction of CM based on ABS and *o*-, *m*-, *p*-carboxyphenylmaleimides.

Composition of the Composites (Mass %)	E_a,_ kJ·mol^−1^	Mass Loss, %								
		Temperature, °C								
		300	325	350	375	400	420	440	450	475
*o*-CPhMI/ABS	179	0	2	19	20.1	25	74.5	75.5	86.8	87
*m*-CPhMI/ABS	125	0	0	3	3	7.8	27	82	84	95.5
*p*-CPhMI/ABS	112.2	0	1	1.5	6	10	26	75	79	99.1

## Data Availability

The original contributions presented in this study are included in the article. Further inquiries can be directed to the corresponding author(s).

## Abbreviations

The following abbreviations are used in this manuscript:

ABS	acrylonitrile–butadiene–styrene
BAAs	biologically active additives
CM	composite materials
CPhMI	carboxyphenylmaleimide
DTA	differential thermal analysis
TGA	thermogravimetric analysis
IR	Infrared Spectroscopy
UV	ultraviolet spectroscopy
*S. aureus*	*Staphylococcus aureus*
*P. aeruginosa*	*Pseudomonas aeruginosa*
*E. coli*	*Escherichia coli*
*C. albicans*	*Candida albicans*
